# Primary Intracranial Gliosarcoma: Is It Really a Variant of Glioblastoma? An Update of the Clinical, Radiological, and Biomolecular Characteristics

**DOI:** 10.3390/jcm13010083

**Published:** 2023-12-22

**Authors:** Domenico La Torre, Attilio Della Torre, Erica Lo Turco, Prospero Longo, Dorotea Pugliese, Paola Lacroce, Giuseppe Raudino, Alberto Romano, Angelo Lavano, Francesco Tomasello

**Affiliations:** 1 Department of Medical and Surgery Sciences, School of Medicine, AOU “Renato Dulbecco”, University of Catanzaro, 88100 Catanzaro, Italy; a.dellatorre@unicz.it (A.D.T.); lngpsp97b07a225j@studenti.unime.it (P.L.); paola.lacroce@studenti.unime.it (P.L.); lavano@unicz.it (A.L.); 2 Humanitas, Istituto Clinico Catanese, 95045 Catania, Italy; dorotea.pugliese@humanitascatania.it (D.P.); giuraudino@hotmail.it (G.R.); alberto.romano@ccocatania.it (A.R.); ftomasel@unime.it (F.T.)

**Keywords:** primary gliosarcoma, overall survival, glioblastoma, IDH, MGMT, hTERT

## Abstract

Gliosarcomas (GS) are sporadic malignant tumors classified as a Glioblastoma (GBM) variant with IDH-wild type phenotype. It appears as a well-circumscribed lesion with a biphasic, glial, and metaplastic mesenchymal component. The current knowledge about GS comes from the limited literature. Furthermore, recent studies describe peculiar characteristics of GS, such as hypothesizing that it could be a clinical–pathological entity different from GBM. Here, we review radiological, biomolecular, and clinical data to describe the peculiar characteristics of PGS, treatment options, and outcomes in light of the most recent literature. A comprehensive literature review of PubMed and Web of Science databases was conducted for articles written in English focused on gliosarcoma until 2023. We include relevant data from a few case series and only a single meta-analysis. Recent evidence describes peculiar characteristics of PGS, suggesting that it might be a specific clinical–pathological entity different from GBM. This review facilitates our understanding of this rare malignant brain tumor. However, in the future we recommend multi-center studies and large-scale metanalyses to clarify the biomolecular pathways of PGS to develop new specific therapeutic protocols, different from conventional GBM therapy in light of the new therapeutic opportunities.

## 1. Introduction

Gliosarcoma (GS) was first described by Strӧebe in 1895, but its acceptance and complete understanding developed later thanks to the detailed description provided by Feigen and Gross in 1955. They were the first to recognize three malignant brain tumors composed of two different tissues: one of glial origin, similar to Glioblastoma, and the other of mesenchymal origin, with characteristics reminiscent of spindle cell sarcomas [1,2,3]. In the 2000 World Health Organization (WHO) Classification, GS was first recognized as a variant of GBM [4]. In 2016 and 2021, WHO successfully classified GS as a variant of GBM with IDH-wild type phenotype [5,6]. Effectively, the radiological, biomolecular, and clinical features reported in the literature about GS are similar to those of GBM. GS is described as a rare form of neoplasm with an inferior prognosis [7]. Its incidence varies between 1% and 8% of all malignant gliomas, representing only 0.48% of all brain tumors and from 1.8% to 2.8% of cases of GBM [2,7,8,9]. GS are most common in adults, with a median age of diagnosis of 60 years, with a male predilection (M:F 1.8:1). In pediatric individuals, it is scarce. With regard to ethnicity, it is more frequent in the white and non-Hispanic races [1,2,8,10,11]. This type of cancer can occur in both primary and secondary forms, with the latter thought of arising from previously treated GBM. From a therapeutic point of view, the commonly used strategy is the same adopted for GBM, or the Stupp protocol, which involves the administration of TMZ concomitantly with RT [2,12,13]. Nevertheless, without any treatment, the prognosis of GS is inferior, with a median survival of approximately four months [9]. While with standard treatments, survival for GS remains still poor, with a median survival of 9 months, compared to other forms of GBM associated with an average of 15 months survival [9,14,15]. Moreover, the most recent literature suggests that GS may have neuroradiological, histological, and biomolecular characteristics that differ from GBM [8,11,16]. Given ongoing debate and uncertainty, we conducted an updated systematic review of the relevant literature to evaluate the possibility that GS may be a distinct entity from GBM, with its own peculiar radiological, biomolecular, and clinical patterns, to push research to develop more specific and effective treatments able to improve overall survival (OS).

## 2. Materials and Methods

### 2.1. Protocol, Search Strategy, and Study Selection

**Figure 1:**
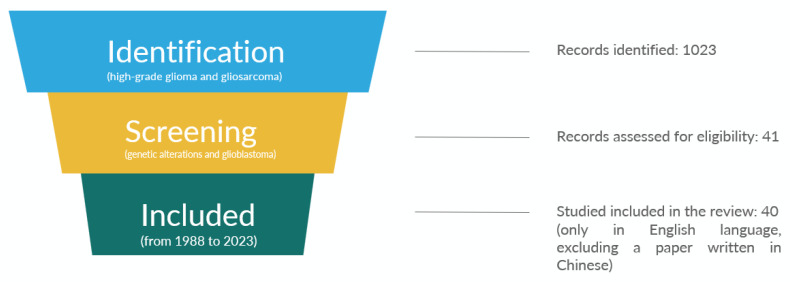
PRISMA flow diagram of included studies.

The systematic review was performed per the Preferred Reporting Items for Systematic Reviews and Meta-Analysis (PRISMA) guidelines. At first, a comprehensive literature review of the databases PubMed and Web of Science was conducted over the past 20 years (2013–2023) using search terms relevant to the different topics: “(high-grade glioma [MeSH Terms])”, “(gliosarcoma [MeSH Terms]) or (genetic alterations [MeSH Terms])” combined with “globlastoma [MeSH Terms])”, including articles focused on gliosarcoma until 2023. Subsequently, given the small number of articles published in GS and the relatively few cases reported per study, all manuscripts published between 1988 and 2023 were considered. Therefore, we identified 1023 manuscripts. Among these, after reading the title and abstract, we assessed the eligibility of 41 studies. One of these documents was later excluded because it was written in Chinese. Ultimately, we included 40 relevant studies, all written in English. (Figure 1) Summary of all the studies included in the systematic literature review are shown in Table 1.

### 2.2. Data Collection and Analysis

After screening and reviewing the studies, we searched and extracted the following information: author, country, journal, title, and year of publication; design and period in which the population was collected; sample size, mean, and age range; genetic and biomolecular data; clinical features, including mild symptoms to more severe conditions; number and percentages of metastases, radiological features, treatment options including surgery, adjuvant radiation therapy (RT), chemotherapy, and other adjuvant therapies; follow-up period; and prognosis and outcome.

## 3. Radiological Features: GS vs. GBM

GS may have some radiological characteristics that can help to distinguish it from GBM. These features include well-demarcated margins, solid-cystic components, the salt and pepper (S–P) sign (a crescent-shaped area of enhancement at the junction of the solid and cystic components), an uneven rim- and a ring-like or paliform enhancement (P-E) patterns enhancement, intra-tumoral strip enhancement, involvement of deep structures such as the thalamus, brainstem, and spinal cord. In addition, GS may also present with other radiological findings, such as midline shift, mass effect, and calcifications [3,11]. However, although they are typical radiological features of GS, similar radiological features can also be observed in several brain tumors, including GBM and high-grade gliomas (HGG) [3,11,55,56].

Yi et al. [55], in their radiological analysis, found that the degree of tumor wall thickening tends to be more significant in GS compared to GBM. Moreover, GS, unlike GBM, seems to have a higher rate of bleeding, S–P signs, an eccentric cystic portion (ECP), and a P-E pattern. In their 48 patients, they found that GS tumors are typically larger than GBM tumors, with more areas of enhancement. Unlike GBM, GS tumors are more likely to involve the brain’s cortex and are less likely to have necrosis, invade the ependyma, and cause edema that crosses the brain’s midline [55]. Moreover, a higher percentage of eccentric tumor cysts in GS was found (19/48, 39.6%) [12].

Zhang et al. [11], in their retrospective single-center study focused on 103 GS, found that 67 tumors were single lesions, and 31 were cystic, solid lesions. All GS showed marked enhancement, and most tumors showed it in functional areas. Notably, 35, 4, 15, 13, and 22 patients showed a pattern of enhancement in the thalamus, brainstem, motor available cortex, sensory functional cortex, and the ependyma of the lateral ventricle, respectively. On T2WI MRI sequences, the average edema diameter was calculated at 7.90 cm (range, 3.55–12.88 cm), and the median tumor diameter evaluated by contrast-enhanced T1WI was 4.84 cm (range, 1.58–8.73 cm) [11]. Tumors involved the frontal, parietal, temporal, or multiple lobes in 18, 6, 29, and 40 patients. While only in 5 patients, the tumors were located in different areas (thalamus, ventricle, brainstem, and spinal cord). Similar results have been reported by Xi et al. [55]. In their series of 48 patients, GS was mainly located in the temporal lobe (27%), frontal lobe (17%), and ventricles (10%), while more rarely in the parieto-occipital lobes (2%), brainstem, and cerebellum (2%). Regarding the laterality, the right hemisphere is mainly affected [55].

Aya Fukuda et al. [57], in their report of three patients, described that at the CT scan, GS typically appears as an expansive lesion with well-delimited and irregular contours, associated with perilesional edema with a frequent hyperattenuating sign of the solid part. Regarding MRI on the T1- and T2-weighted sequences of MRI, GS were characterized as uneven, heterogeneous tumors correlated with bleeding at distinct stages with a hypo-isointense on T1 and as hypo/iso/hyperintense on T2 of the solid part. Similarly, the necrotic part was described as hypointense on T1 and hyperintense on T2. Inhomogeneous enhancement of the solid components occurred after the injection of gadolinium. The SWI or T2* sequence supplied other information; the variable magnetic susceptibility (high heterogeneity) areas showed hypointensity within the tumor due to bleeding or newly formed vessels/flow voids. On DWI/ADC mapping sequences, GSM has previously been associated with hyperintensity on DWI and hypointensity in the solid component on the ADC map (compatible with restricted diffusion) [57].

Han et al. [58]. classified two different subgroups of patients: one with tumors that resembled the characteristics of meningioma (meningioma-like) and the other that mimicked the appearance of GBM (GBM-like). The meningioma-like tumors displayed significant rim enhancement on MRI, and more of them demonstrated homogeneous enhancement compared to the GBM-like sub-group [58]. However, these findings were not found to be statistically significant [58]. Results are summarized in Table 2.

## 4. Genetics and Biomolecular Patters: GS vs. GBM

It has been observed that the monoclonal origin of GS would be associated with the p53 mutation, found in 23% of GS compared to 11% of primary GBM, and the deletion of p16. Epidermal Growth Factor Receptor (EGFR) amplification was only seen in 4% of GS compared to 35% of GBM [2,3,59,60].

There were slight differences between GBM and GS in Phosphatase and Tensin homolog (PTEN) mutations and Cyclin-dependent kinase (CDK) amplification found in both glial and sarcomatous components [61]. In addition, less than 12% of GS have methylation of the O6-methylguanine-DNA methyltransferase gene promoter (pMGMT), which is associated with a good prognosis [11].

From a biomolecular point of view, GS has mutations in common with soft tissue sarcoma due to involvement in the promoter of the Telomerase reverse transcriptase gene (pTERT), Tumor Protein 53 (TP53), Neurofibromin 1 (NF1), Cyclin-dependent kinase inhibitor 2A (CDKN2A), Cyclin-dependent kinase inhibitor 2B (CDKN2B) and Retinoblastoma associated Protein Type 1 (RB1) [60,62]. Similarly, to GBM, GS shows mutations in PTEN, EGFR, Stromal Antigen 2 (STAG2), and Protein Tyrosine Phosphatase Non-Receptor Type 11 (PTPN11) [7,9,11].

Sarcomatous-predominant GS has several features similar to meningioma. It is characterized by positivity to reticulin and the absence of GFAP expression, while predominant gliomatous GS has characteristics reminiscent of GBM, such as necrosis, lack of reticulin production, and GFAP positivity [8].

Zaki et al., in their study, compared common gene alteration, greater than 5%, in GS, GBM, and soft tissue sarcoma. Among these, GS shared only four genes with GBM, none with sarcomas, while nine common genes were found unique for GS amongst the 5% threshold for each respective tumor type [2]. They concluded that most of these mutations overlap with GBM and other cancers; nevertheless, GS has its own genetic mutations, such as MutS Homolog 6 (MSH6), B-Raf proto-oncogene serine/threonine kinase (BRAF), Suppressor of Zeste 12 (SUZ12), Sex Determining Region Y Box Transcription Factor 2 (SOX2), and Box and WD Repeat Domain Containing 7 (FBXW7) [2,7,11,16,60].

Nevertheless, it has been previously reported that, BRAF V600E mutation, SOX2 amplifications, and MSH6 mutation are present approximately in 3%, 10% and 20% of GBMs, respectively [16,63]. Results are summarized in Table 3.

## 5. Clinical Features and Behavior

### 5.1. Clinical Characteristics

Han et al. [58] observed that clinical manifestations of GS are not specific. Still, it can manifest with intracranial hypertension syndrome characterized by symptoms ranging from headache, projectile vomiting, and hemiparesis up to more severe conditions such as the state of drowsiness and, finally, coma [58]. This symptomatology is due to the mass effect given by the tumor and the extensive peri-lesional edema or acute, intra-lesional, or more rarely peri-lesional symptomatic intracranial bleeding [11,58]. Other symptoms are asthenia, personality disorders, and mental confusion [10,58]. Moreover, depending on the site in which the tumor occurs, it can lead to different neurological deficits: language disorder (dysphasia, aphasia), sensory alterations, paresis of a part of the body, decreased visual acuity, and campimetric deficit [1,10].

### 5.2. Metastases

Saadeh et al. [9] observed that extracranial metastases from GS tend to be more frequent than from GBM and other malignant brain tumors, in which they are sporadic. Indeed, extracranial metastases were reported in 11% (range 0–16%) of GS, mainly including the lungs, liver, and lymph nodes, 72%, 41%, and 18%, respectively. While, more rarely, metastases occur in the spleen, adrenal glands, kidneys, oral mucosa, skin, bone marrow, skull, ribs, and spine [1,9,58,64].

Other organs affected may be the thyroid, pericardium, myocardium, diaphragm, pancreas, and stomach [1,9,58].

Moreover, it has been reported that metastatic foci of GS may have both gliomatous and sarcomatous components [9]. However, recent studies reported that the sarcomatous component was mainly represented. These findings may suggest that the sarcomatous component of GS is more likely to metastasize and disseminate by the hematogenous route than its gliomatous counterpart [9,10,58].

The development of metastasis from GS is established through numerous case reports, and the rate of metastasis found in the literature is about 11%. Despite the rarity of PGS, these reports support the clinical experience that GS may have a more significant potential for metastasis than GBM [9,65]. Indeed, it has been suggested that due to the higher resistance of GS to current treatment compared to GBM, malignant cells that are not destroyed might become more aggressive, metaplastic, and, thus, angio-invasive [9,66].

## 6. Treatment and Prognosis

### 6.1. Surgical Strategy

The prognosis of GS is inferior, with a median survival of approximately four months without any treatment [9,14].

To date, no specific treatment for GS has been developed. Currently, standard GBM treatment is adopted for GS patients with good Karnofsky Performance Status (KPS) [16,67,68]. However, the most recent literature shows that GS presents different response patterns to therapies than GBM, thus hypothesizing that GS might be a different clinical–pathological entity [8,16,66].

Indeed, a Maximal Safe Resection (MSR) associated with a concomitant Radio- and Adjuvant Chemotherapy (CCRT) reduces the mortality rate in both cancers. Still, the response to treatments seems to be different in GS [12,13]. The peculiar biphasic, glial, and metaplastic mesenchymal components of GS might explain it.

Gross Total Resection (GTR) or Subtotal Resection (STR) when resection involves >90% but <100% of tumor tissue or biopsy are standard surgical treatments for GS [12,13]. GTR should be the option of choice. Nevertheless, GTR is almost always possible only in meningioma-like forms, while STR is often performed for GBM-like forms due to its invasive and infiltrative nature. In some cases, due to the location and extent of the lesion, the only viable strategy is stereotactic biopsy [9,12].

Therefore, the higher survival rate of meningioma-like tumors can be attributed to the higher GTR rate in this subtype, which correlates with OS. Due to its characteristics that mimic meningiomas, Sarcomatous GS appears well-delimited to the brain parenchyma; therefore, radical surgical resection is often possible. On the contrary, gliomatous GS, which usually infiltrates, even extensively, the surrounding parenchyma, makes radical excision much more challenging, so it is mainly treated with a STR surgery or biopsy [9].

Unlike GBM, 5-ALA (5-aminolevulinic acid) staining during fluorescence-guided surgery (FGS) in GS tends to assume a heterogenic fluorescence pattern, probably due to its biphasic component [12,16]. However, its role is still being studied [13].

Postoperative complications in GS surgery are similar to those of GBM, including transient or permanent neurological deficits, CSF fistula, surgical focus bleeding, seizure, stroke, and meningitis [12,58,66,69].

### 6.2. Radiotherapy

Only a few studies have evaluated radiotherapy’s (RT) effectiveness in treating patients with GS [12,66]. A significant increase in OS has been observed with surgery followed by RT, which offers a higher outcome (8–15 weeks longer) than surgery alone [12]. Perry et al. confirmed this finding because, in their analysis, 25/32 patients treated with adjuvant radiotherapy had a higher survival rate (46 vs. 13 weeks; *p* = 0.025) [70]. Similar results were found in a study conducted by Castelli et al. [14]. Radiation therapy includes adjuvant external beam radiation therapy (EBRT) and Gamma Knife adjuvant radiosurgery [71]. The standard dose administered is 60 Gray (Gy) in 30 fractions, or another option may be hypofractionated radiation at 40 Gray (Gy) in 15 fractions [13,14,67]. Kozak et al. [7]. investigated the efficacy of radiotherapy in a large cohort of GS patients. In their study, the authors demonstrated that age, extent of resection, and adjuvant radiotherapy (RT) were the most significant predictors of OS. However, the metastatic potential of heavily irradiated tumors needs still to be further investigated. Finally, although the addition of chemotherapeutic agents does not appear to increase OS, it has been theorized that a higher dosage of chemotherapy could still increase survival in patients with GS compared to radiotherapy and surgery alone [14].

### 6.3. Bevacizumab

Bevacizumab, a recombinant monoclonal antibody targeting VEGF receptors on endothelial cells, has demonstrated significant anti-tumor activity in various colon, breast, pancreas, and prostate cancers [72]. Its potential in GBM, a highly vascularized tumor known to produce pro-angiogenic factors, was recognized [73]. Bevacizumab is thought to work by inhibiting the growth of new blood vessels that supply the tumor with oxygen and nutrients. This can lead to tumor shrinkage and a slowing of tumor growth. Bevacizumab can also reduce tumor-related edema, which can improve neurological symptoms [72]. Given the rationale that bevacizumab could hinder GBM and the progression of GS, it was administered to patients with primary gliosarcoma (PGS) and secondary gliosarcoma (SGS). PSG patients who received bevacizumab had improved progression-free survival (PFS) and OS of 4.2 and 8.4 months, respectively, at diagnosis [1]. SGS patient had a PFS of 3.8 months and an OS of 7.3 months [1]. Although the improved outcomes observed in these patients could be attributed to bevacizumab, particularly in recurrent GS, it is also possible that the study population, coming from a referral hospital and already enrolled in clinical trials, may have influenced the results.

### 6.4. Chemotherapy

Various chemotherapeutic agents have been used, and numerous researchers have studied the role and effectiveness of chemotherapy in treating patients with GS [12,13,59,64,74]. Although some studies have presented negative results, others could shed light on the benefits of specific chemotherapeutic agents. Over the years, various agents have been used, such as mitramycin (inhibitor of RNA synthesis), carmustine, administrated alone or together with other systemic agents such as diaziquone, mitomycin C, 6-mercaptopurine and cisplatin), and nitrosureas. These agents, whether used individually or in combination with each other or with radiotherapy, did not appear to have efficacy, either for GBM or GS.

### 6.5. Temozolamide (TZM)

TMZ is an effective treatment in malignant gliomas and still represents the most used chemotherapy drug to manage these tumors. However, although some studies have demonstrated the efficacy of TMZ in treating GS, its role as an effective treatment for GS is still debatable [7,9,12,13].

Indeed, while several studies have reported that TMZ may increase overall survival in patients with GS, others have documented no benefit in prognosis [9,12,14,66]. In their research, Castelli et al. recorded that TMZ, in addition to radiotherapy, effectively increases OS in GBM treatment but not in GS [14]. These findings may be due to the different MGMT methylation of GS compared to GBM. Indeed, GS has a lower rate of MGMT methylation compared to GBM, and this might explain the poor therapeutic response of GS to TMZ [14]. This hypothesis is also confirmed by Kang et al., who demonstrated that GS patients with MGMT methylation had more prolonged overall survival when treated with TMZ [75].

### 6.6. Immunotherapy

Immunotherapy for recurrent GBM, including patients with GS, has been addressed in a few trials. A phase II clinical trial (NCT02798496: CAPTIVE/KEYNOTE-192) evaluated the combination of DNX-2401, an oncolytic adenovirus, with the anti-PD-1 antibody pembrolizumab in patients with recurrent GBM or GS. In this trial, DNX-2401 is delivered directly inside the tumors by intravenous administration of pembrolizumab every three weeks for up to 2 years or until disease progression. Interim data from 42 patients showed a median OS of 12.3 months. This is favorable compared with the OS observed for standard-of-care agents lomustine and temozolomide, which had a median OS of 7.2 months. Four patients survived more than 23 months, and 11.9% (5/42) had durable responses. No dose-limiting toxicities were observed, and adverse events were mild to moderate and unrelated to DNX-2401 [76,77]. However, in the CAPTIVE study, 48 patients with histopathological diagnosis of GBM and only one gliosarcoma (2%) were enrolled; therefore, it is not possible to conclusively argue that there is a different therapeutic response between GBM and GS.

### 6.7. Combined Therapy

Summarizing the findings reported in the reviewed literature, treatment based on Gross Total Resection (GTR), followed by radio- and chemotherapy (TMZ), leads to an increased outcome compared to the single treatment (on average 8–10 months), while no improvements were seen between the dual therapy (TMZ + RT) and monotherapy (TMZ or RT) [9,10].

Castelli et al., in a large series of patients who were treated with a combination of surgery, TMZ, and radiotherapy, reported an average OS of 13 months, and 12% of patients achieved a 2-year OS [14].

Furthermore, Kozak et al. said similar results, showing a significant benefit in the prognosis of GS patients when treated with the multimodal approach. In their study, the authors demonstrated that tumor resection (not just biopsy) and adjuvant RT correlated with increased OS [7].

### 6.8. Prognosis and Outcome

GS owns various prognostic factors that differ from its parent tumor. Older patient age, poorer preoperative clinical status, larger tumor diameter, and tumor location in midline or infratentorial structures were independently associated with shorter OS in the GS cohort [78]. Age and clinical performance are known survival factors in both GS and GBM. The extent of resection (EOR) was not a prognostic factor in the GS cohort [79]. This finding contradicts the convincing data from GBM studies demonstrating the significant role of EOR on patient outcomes. This difference may be due to the small sample size of GS patients [11]. Furthermore, no independent association was found between combined RTX/CTX and GS prognosis. This finding may also be related to the lower MGMT promoter methylation rate in GS. Some studies have also reported lower MGMT promoter methylation rates in GS [11,64]. This difference between GS and GBM may contribute to the limited response of GS to combined treatment with CTX/RTX and TMZ. Other known outcome factors, such as age, preoperative clinical status, and RTX/CTX coadministration, were confirmed to be an independent predictor of survival [31,67].

## 7. Discussion

GS has long been considered a variant of GBM [4,6]. Still, according to our findings, some clinical, radiological, and biomolecular characteristics appear more frequent in GS than in GBM, thus hypothesizing the possibility of underlying differences between these two pathologies [13,16,31] (see Table 4). Analysis of the literature revealed that there were no differences between the two cancers regarding clinical characteristics, age, gender, and preoperative clinical status [31,58]. GS can be characterized by specific radiological features including well-demarcated margins, solid-cystic components, the salt and pepper sign (a crescent-shaped area of enhancement at the junction of the solid and cystic components), an uneven rim- and a ring-like or paliform enhancement (P-E) patterns enhancement, intra-tumoral strip enhancement, and involvement of deep structures such as the thalamus, brainstem, and spinal cord, but all these features may also be found in other malignant brain tumors, including GBM and high-grade gliomas [3,11,55]. Moreover, an eccentric cyst seems to be independently associated with the diagnosis of GS [12]. These typical radiological characteristics of GS may help to distinguish it from GBM.

Interestingly, recent data concerning biomolecular characteristics of GS documented that, although GS has a genetic profile that overlaps with GBM and other neoplasms, it is also true that GS has its genetic mutations, such as MSH6, BRAF, SUZ12, SOX2, and FBXW7 [2,3,10,11,16,80].

Nevertheless, as reported in the literature, BRAF V600E mutation is present in 10% of GSs, compared to 3% of GBMs, while amplifications of the SOX2 gene and MSH6 mutation are present approximately in 10% and 20% of GBMs, respectively [16,63]. However, Zaki et al., in their recent study, reported that BRAF mutations (G32_A33duo, G466E, V600E protein alteration), MSH6 mutations (L1244dup, T1133A protein alteration), and SOX2 amplification (11% alteration frequency), are unique to GS [2].

This apparent contradiction could be due to the fact that in their study, Zaki et al. considered as common genetic alterations only those genes that were altered in more than 5% of the samples analyzed for each tumor type, with a minimum of genetic alteration in >2 samples. Therefore, although with some concerns, these specific biomolecular mutations could partially explain the different biological behavior, response to therapy, and prognosis of GS compared to GBM [9,14].

Previous studies have vaguely reported survival rates in patients with GBM and GS. While some studies did not find a significant difference in survival between the two tumors, others found a worse prognosis in patients with GS [14,15]. To some extent, heterogeneous landscapes with different distributions of genetic alterations in GBM and GS could explain these discrepant previous findings. In a multivariate analysis, histological diagnosis of GS was associated with a worse prognosis, independent of age, preoperative KPS, EOR, and postoperative treatment. This association is due to lower MGMT promoter methylation rates and lower frequency of IDH1 mutations in the GS cohort [7,13,81]. Indeed, after including only IDH1 wild-type patients in the analysis and MGMT promoter methylation, it was found that the histological diagnosis of GS was no longer associated with worse outcomes [9]. Furthermore, lower levels of GFAP and higher levels of TP53 staining predicted GS diagnosis [3,7,10].

Unlike GBM, GS appears to have a greater propensity to metastasize outside the central nervous system. Based on older studies, until 2007, it has been estimated that the frequency of metastases varies between 0.4% and 2.0%. However, the only two systematic reviews summarizing results published up to 2008 are partly conflicting; therefore, many relevant questions remained unanswered, including the rate of extracranial metastases. On the other hand, the available literature on this issue mainly reported that GSs are more prone to extracranial metastasis than GBM [1,9,58,64]. Furthermore, a recent meta-analysis including ten studies published between 2008 and 2018 said that extracranial metastases in GS were up to 11% and significantly higher than in GBM (11% versus 0.2–4.0%, respectively) [12]. Nevertheless, considering data reported in the available literature, the percentages of extracranial metastasis ranged from 0 to 16%.

From a therapeutic point of view, the literature data are speculative and inconclusive. Currently, the Stupp protocol is widely recommended for GS patients in clinical settings, involving radiotherapy and chemotherapy following surgery GBM. However, in GS, the response to the therapy is variable and different if compared to those of GBM. Radiotherapy has been proposed to enhance patient outcomes, as it can extend overall survival by 2–4 months [9,15,78,79]. TMZ still represents the most effective drug for malignant gliomas [67]. Despite this, there is an ongoing debate about the therapeutic benefits of RT and TMZ in GS, as there is no prospective or large scale analysis. It should stimulate further research into GS-targeted therapies.

## 8. Conclusions and Future Directions

Overall, the present review supports the hypothesis that GS is a rare yet devastating tumor with specific imaging, immunohistochemical, and clinical features that are more likely to occur when compared to GBM. This raises the possibility of distinguishing this disease from other malignant brain neoplasms. To date, the standard treatment for GS is similar to that most used to treat GBM, which involves surgery associated with adjuvant therapy, including RT, chemotherapy alone or in combination. It has been shown that maximum safe resection followed by radio and chemotherapy (TMZ) leads to a better outcome than a single treatment.

GTR (when possible) should be the option of choice among other surgical procedures, including subtotal resection (STR) or biopsy. On the other hand, different published studies documented that EOR was not a prognostic factor in GS patients. On the contrary, credible data from GBM studies demonstrate the significant role of EOR on patient outcomes. We believe that, similarly to other malignant brain tumors, GTR reduces the mortality rate in GS. But, due to the small sample size of patients, the peculiar biphasic, glial, and metaplastic mesenchymal, which sometimes makes it challenging to achieve a GTR, and the different response to treatments of GS compared to GBM may explain this apparent contradiction. Nevertheless, GS’s prognosis is poorer than GBM’s, and the optimal treatment for this rare neoplasm remains speculative. Moreover, we need more extensive prospective studies to evaluate new specific treatment regimens. It should stimulate further research into GS-targeted therapies. The results of the CAPTIVE/KEYNOTE-192 trial are promising but not definitive. However, it could open up possible future scenarios for developing effective and safe treatments for GS [76,77]. With some limitations, mainly due to the scarcity of data and the rarity of this tumor, which limits the relevant literature on the topic, this review could represent a valid background for designing future studies better to describe the characteristics of this rare and dismal malignancy. Therefore, we recommend multi-center studies and large-scale metanalyses to better elucidate typical features of GS, thus hypothesizing specific treatment regimens.

## Figures and Tables

**Table 1:** Summary of studies included in the systematic literature review.

No.	Author, Journal, Year	Title	Type of Study	Study Period	Sample Size	Area of Interest
1	Oh et al. 2016 [17]	Genetic Alterations in Gliosarcoma and Giant Cell Glioblastoma.	Case series	N/A	55	Biomolecular
2	Saadeh et al. 2019 [9]	Prognosis and management of gliosarcoma patients: A review of literature.	Review	Up to 2019	N/A	Characteristic, prognosis and management
3	Tauziède-Espariat et al. 2018 [18]	Cerebellar high-grade gliomas do not present the same molecular alterations as supratentorial high-grade gliomas and may show histone H3 gene mutations.	Retrospective study	1982–2016	19	Biomolecular
4	Li et al. 2021 [19]	Genetic alteration and clonal evolution of primary glioblastoma into secondary gliosarcoma.	Case Report	2016	1	Biomolecular
5	Esteban-Rodríguez et al. 2023 [20]	Cytological features of diffuse and circumscribed gliomas.	Review	N/A	N/A	Biomolecular
6	Sahu et al. 2022 [21]	Rat and Mouse Brain Tumor Models for Experimental Neuro-Oncology Research.	Review	N/A	N/A	Characteristics and biomolecular
7	Zaki et al. 2021 [2]	Genomic landscape of gliosarcoma: distinguishing features and targetable alterations.	Scientific Reports	N/A	30	Biomolecular
8	Kleihues et al. 2000 [22]	Phenotype vs. genotype in the evolution of astrocytic brain tumors.	Case series	N/A	N/A	Genetics and biomolecular
9	Wang et al. 2017 [23]	Gliosarcomas with the BRAFV600E mutation: a report of two cases and review of the literature.	Case report	N/A	2	Biomolecular
10	Bax et al. 2009 [24]	EGFRvIII deletion mutations in pediatric high-grade glioma and response to targeted. therapy in pediatric glioma cell lines.	Retrospective study	N/A	90	Biomolecular
11	Reis et al. 2000 [25]	Genetic profile of gliosarcomas.	Short communication	N/A	19	Genetics and biomolecular
12	Cheng et al. 2022 [26]	Gliosarcoma: The Distinct Genomic Alterations Identified by Comprehensive Analysis of Copy Number Variations.	Retrospective study	2016–2019	36	Genetics and biomolecular
13	Lowder et al. 2019 [27]	Gliosarcoma: distinct molecular pathways and genomic alterations identified by DNA copy number/SNP microarray analysis.	Metanalysis	2014–2015	18	Genetics and biomolecular
14	Codispoti et al. 2014 [28]	Genetic and pathologic evolution of early secondary gliosarcoma.	Case report	N/A	1	Genetics and biomolecular
15	Anderson et al. 2020 [29]	Molecular and clonal evolution in recurrent metastatic gliosarcoma.	Case report	N/A	1	Characteristics and biomolecular
16	Garber et al. 2016 [30]	Immune checkpoint blockade as a potential therapeutic target: surveying CNS malignancies.	Retrospective analysis	2009–2016	347	Biomolecular and prognosis
17	Pierscianek et al. 2021 [31]	Demographic, radiographic, molecular and clinical characteristics of primary gliosarcoma and differences to glioblastoma.	Retrospective cohort study	2001–2018	56	Clinical, prognosis and neuroradiological features
18	Walker et al. 2001 [32]	Characterisation of molecular alterations in microdissected archival gliomas.	Retrospective analysis	N/A	47	Genetics and biomolecular
19	Hiniker et al. 2013 [33]	Gliosarcoma arising from an oligodendroglioma (oligosarcoma).	Case report	N/A	1	Biomolecular, clinical
20	Dejonckheere et al. 2022 [34]	Chasing a rarity: a retrospective single-center evaluation of prognostic factors in primary gliosarcoma.	Retrospective study	1995–2021	26	Clinical features, treatment and prognosis
21	Chen et al. 2022 [35]	Gliosarcoma with osteosarcomatous component: A case report and short review illustration.	Case report+ Review	1950–2022	13	Biomolecular, neuroradiology, treatment and prognosis
22	Nagaishi et al. 2012 [36]	Amplification of the STOML3, FREM2, and LHFP genes is associated with mesenchymal differentiation in gliosarcoma.	Case series	N/A	74	Biomolecular
23	Boerman et al. 1996 [37]	The glial and mesenchymal elements of gliosarcomas share similar genetic alterations.	Case series	N/A	5	Genetics and biomolecular
24	Schwetye et al. 2016 [38]	Gliosarcomas lack BRAFV600E mutation, but a subset exhibit β-catenin nuclear localization.	Case series	N/A	48	Biomolecular
25	Cho et al. 2017 [39]	High prevalence of TP53 mutations is associated with poor survival and an EMT signature in gliosarcoma patients.	Comparative analyses	N/A	103	Biomolecular
26	Actor et al. 2002 [40]	Comprehensive analysis of genomic alterations in gliosarcoma and its two tissue components.	Comprehensive analysis	N/A	38	Genetics and biomolecular
27	Sargen et al. 2023 [41]	Estimated Prevalence, Tumor Spectrum, and Neurofibromatosis Type 1-Like Phenotype of CDKN2A-Related Melanoma-Astrocytoma Syndrome.	Retrospective cohort study	1976–2020	640 292	Genetics and biomolecular
28	Gondim et al. 2019 [42]	Determining IDH-Mutational Status in Gliomas Using IDH1-R132H Antibody and Polymerase Chain Reaction.	Case series	N/A	62	Biomolecular
29	Reis et al. 2005 [43]	Molecular characterization of PDGFR-alpha/PDGF-A and c-KIT/SCF in gliosarcomas.	Case series	N/A	160	Biomolecular
30	Tabbarah et al. 2012 [44]	Identification of t(1;19) (q12;p13) and ploidy changes in an ependymosarcoma: a cytogenetic evaluation.	Case report	N/A	1	Genetics and biomolecular
31	Knobbe et al. 2003 [45]	Genetic alterations and aberrant expression of genes related to the phosphatidyl-inositol-3’-kinase/protein kinase B (Akt) signal transduction pathway in glioblastomas.	Comparative Study	N/A	103	Genetics and biomolecular
32	Bigner et al. 1988 [46]	Specific chromosomal abnormalities in malignant human gliomas.	Case series	1981–1986	54	Genetics and biomolecular
33	Jimenez et al. 2011 [47]	Sarcoma arising as a distinct nodule within glioblastoma: a morphological and molecular perspective on gliosarcoma.	Case report	N/A	1	Biomolecular
34	Albrecht et al. 1993 [48]	Distribution of p53 protein expression in gliosarcomas: an immunohistochemical study.	Case series	N/A	8	Biomolecular
35	Lusis et al. 2010 [49]	Glioblastomas with giant cell and sarcomatous features in patients with Turcot syndrome type 1: a clinicopathological study of 3 cases.	Case report	1996–2010	3	Biomolecular
36	Visani et al. 2017 [50]	Non-canonical IDH1 and IDH2 mutations: a clonal and relevant event in an Italian cohort of gliomas classified according to the 2016 World Health Organization (WHO) criteria.	Multicenter study	N/A	288	Genetics and biomolecular
37	Barnett et al. 2004 [51]	Intra-arterial delivery of endostatin gene to brain tumors prolongs survival and alters tumor vessel ultrastructure.	Prospective study	N/A	344	Genetics, treatment and prognosis
38	Bigner et al. 1988 [52]	Gene amplification in malignant human gliomas: clinical and histopathologic aspects. *J Neuropathol Exp Neurol.*	Retrospective study	N/A	64	Genetics, biomolecular and clinical features
39	Koelsche et al. 2013 [53]	Distribution of TERT promoter mutations in pediatric and adult tumors of the nervous system.	Systematic analysis	N/A	1515	Genetics and biomolecular
40	Venkatraj et al. 1998 [54]	Genomic changes in glioblastoma cell lines detected by comparative genomic hybridization.	Comparative Study	N/A	5	Biomolecular

**Table 2:** Common radiological features in Gliosarcoma (GS) vs. Glioblastoma (GBM).

Radiological Features	Study	Result
Larger wall thickening GS > GBM.	Yi et al. (2018) [55]	Confirmed
Higher rate of bleeding, and S–P sign, presence of eccentric cystic portion (ECP) and a P-E pattern. GS > GBM.	Yi et al. (2018) [55]	Confirmed
Larger tumors with more areas of enhancement. GS > GBM	Yi et al. (2018) [55]	Confirmed
More likely to involve the brain’s cortex. Less likely to have necrosis, to invade the ependyma and to cause edema that crosses the brain’s midline. GS > GBM	Yi et al. (2018) [55]	Confirmed
Higher percentage of eccentric tumor cysts. GS > GBM	Yi et al. (2018) [55]	Confirmed
Marked enhancement, and most of tumors showing it in functional areas. GS > GBM	Zhang et al. (2021) [11]	Confirmed
GS: mainly located in temporal lobe (27%), frontal lobe (17%) and ventricles (10%); while more rarely in the parieto-occipital lobes (2%), brainstem and cerebellum (2%).	Zhang et al. (2021) [11]	Confirmed
Appearance as an expansive lesion with well-delimited and irregular contours, associated with perilesional edema with a frequent hyperattenuating sign of the solid part. GS > GBM	Aya Fukuda et al. (2020) [57]	Confirmed
Association with hyperintensity on DWI and hypointensity in the solid component on the ADC map (compatible with restricted diffusion). GS > GBM	Aya Fukuda et al. (2020) [57]	Confirmed

**Table 3:** Common biomolecular markers in Gliosarcoma (GS) vs. Glioblastoma (GBM).

Biomolecular Markers	GS	GBM	Study
p53 mutation	23%	11%	Saadeh et al. (2019) [9]
			Wojtas et al. (2019) [60]
p16 deletion	37%	No	Saadeh et al. (2019) [9]
			Zaki et al. (2021) [2]
EGFR amplification EGFR mutation	4% No	35% Yes	Romero et al. (2013) [3]
			Zaki et al. (2021) [2]
PTEN mutation	(37%)	Yes	Saadeh et al. (2019) [9]
CDK amplification	Yes	Yes	Dardis et al. (2021) [16]
pMGMT methylation	<12%	Yes	Smith et al. (2018) [10]
pTERT mutation	Yes	Yes	Zaki et al. (2021) [2]
NF1 mutation	Yes	Yes	Zaki et al. (2021) [2]
CDKN2A/B mutation	Yes	Yes	Wojtas et al. (2019) [60]
RB1 mutation	Yes	Less common (~20%)	Wojtas et al. (2019) [60]
STAG2 mutation	Yes	Yes	Wojtas et al. (2019) [60]
PTPN11 mutation	Yes	Yes	Saadeh et al. (2019) [9]
Reticulin positivity	Sarcomatous-predominant GS	No	Han et al. (2010) [58]
GFAP expression	Gliomatosus-predominant GS	Yes	Han et al. (2010) [58]
MSH6 mutation *L1244dup, T1133A*	Yes	No	Zaki et al. (2021) [2]
BRAF mutation BRAF mutations (all alteration types)	10%	3%	Zaki et al. (2021) [2]
	10%	0%	Zaki et al. (2021) [2]
SUZ12 mutation	Yes	No	Zaki et al. (2021) [2]
SOX2 mutation	Yes	No	Zaki et al. (2021) [2]
FBXW7 mutation	Yes	No	Zaki et al. (2021) [2]

**Table 4:** Summary of common features in Gliosarcoma (GS) vs. Glioblastoma (GBM).

Feature	GS	GBM
Clinical presentation	Non-specific; can manifest with intracranial hypertension syndrome	Non-specific; can manifest with intracranial hypertension syndrome
Radiological features	Well-demarcated margins, solid-cystic components, salt and pepper sign, uneven rim- and ring-like enhancement patterns	Irregular margins, necrosis and peritumoral edema
Genetic profile	More likely to have p53 mutations and p16 deletions, less likely to have EGFR amplification and pMGMT methylation	p53 mutations, p16 deletions, PTEN mutations, CDK amplification, EGFR amplification, STAG2 mutations and PTPN11 mutations
Extracranial metastatic potential	More frequent (11%)	Extremely rare
Sites of metastases	Lungs (72%), liver (41%), lymph nodes (18%), spleen, adrenal glands, kidneys, oral mucosa, skin, bone marrow, skull, ribs and spine	N/A
Treatment	Maximum safe surgical resection followed by CCRT	Maximum safe surgical resection followed by CCRT
Prognosis	Worse than GBM	Poor

CCRT: chemo-radiotherapy.

## Data Availability

Not applicable.
